# What It Offers to the Newly-Qualified and Others

**Published:** 1920-09-04

**Authors:** 


### THE MENTAL HOSPITAL SERVICE.

### What It Offers to the Newly-Qualified and Others.

The exuberant optimism of various castes of psychic healers as to the possibilities of their systems eliminating the graver forms of mental disorder may arouse apprehensions on the part of those who are contemplating work in mental hospitals that, ere long, they may find their occupation gone. But all the problems are not going to be settled as easily as some people appear to think they will be; and, therefore, for many years to come there will be work for those who choose psychiatry as their profession. Indeed, it is difficult to foresee a time when there will no longer be the need for the homes for the incurably insane, however complete our therapeutic system may be. And, in spite of the critics, it has not yet been proved that, for cases of acute and curable insanity, the mental hospital is ineffective.

### Financial and Social Prospects.

It may seem to derogate from a position of idealism to consider in the first place the financial question; but in these hard times it is essential. Fortunately it has been slowly realised that in order to attract men to take up this kind of work it is necessary to pay reasonable salaries; and in addition to make it possible for some of them to avoid the celibacy that was the portion of their predecessors. But there still remains much to do. It is true the initial salary is a good one for anyone newly qualified—say, somewhere in the region of £300 with "everything found," or with a corresponding increase of salary if the catering is left to the individual. The increments in salary lead up to a sum of about £500 a year with, in the case of the senior assistant medical officer, "married quarters." In some asylums arrangements have been made for the second A.M.O. to have similar quarters if he desires to marry. For the most part, medical officers' housing compares almost favourably with the accommodation allocated to the other officials, for example, the clerk and steward, or the farm bailiff. The medical superintendent is, however, in a better situation. He has a house and a garden, as a rule; and he is an autocrat whose power is tempered by the decisions of a committee. (He does not express it quite thus himself. He is apt to say that he is but an unhappy mortal, ground between upper and nether mill-stones in the shape of committees and commissioners—with much lateral pressure also from relatives and friends of patients!)

### Looking Ahead.

It is, however, wise to look ahead if the intention is to take up this branch of the profession as a life-work. It will then be seen that the scope for

**The Mental Hospital Service**—(*continued*).

initiative is limited. The number of medical superintendents is relatively small. In any case such a position will only be attained, as a rule, by seniority. Even that exalted state does not compare very favourably with what may be achieved in other specialities. A salary of £1,000 to £1,200 a year, even with emoluments, would not attract the medical or surgical specialist; even the successful practitioner might be inclined to look askance at it. Apart, however, from the actual sum which can be earned there is the deadening of initiative resulting from the fact that, no matter how much work, scientific or otherwise, is done there is not any corresponding advancement.

### An Assured Income.

On the other hand, for those who are content with a moderate but assured income there are no worries in regard to the question of an annual holiday and the leaving of a *locum tenens* to carry on—or dissipate—one's practice; and very little in the way of night-work, no long journeys in all weathers, and on the whole nothing unduly strenuous to be faced in the daily routine. Also, asylums are, for the most part, pleasantly situated and there is a social circle of which the medical officer may be an important unit. The more social accomplishments he has the better. To be able to take part in games, to be able to dance, or to sing or to play, any of these serve to render him a more acceptable, happier member of the institutional community. It is these things, too, allied with kindness, consideration, sympathy, which really give rise to the "atmosphere" which counts for so much, even from a therapeutic point of view.

### Opportunities for Study.

For those who are interested in the scientific aspect of psychiatry there is plenty of opportunity for study. There is a wealth of clinical material, and, especially in the country asylums, it is possible to do pathological research. Unfortunately in this direction also so very much depends on the individual; and early enthusiasm is apt to be damped when encouragement is lacking. Still, even in this direction, there are signs of grace. Suggestions are being made for study-leave, to enable medical officers to keep in touch with the latest advances in research and to work for the special examinations of the diploma in psychological medicine.

The work in the private mental hospitals is very much the same from the clinical point of view as in the public ones and the chances of advancement are about equally exiguous. Pay is for the most part on similar levels. But there is a difference in regard to much of the routine work; there is more to be done in the matter of arranging for the admission of cases, in interviewing relatives and friends, and in cultivating the social side. On the other hand, there is a greater opportunity of meeting cultured and interesting people among visitors and patients. There is also possibly the opportunity of doing a certain amount of consultative work, especially in the private asylums situated in or near towns, and eventually a number of men engaged in both private and public work gravitate in this direction. There are to be taken into account also the lectureships in mental disorders at the various medical schools.

A limited number of appointments for those interested in that particular line of work are those in the State Criminal Lunatic Asylums. These are under the ægis of the Home Office and the holders of them are Government officials. This may be an inducement to those with a penchant for investigating the psychology of the criminal lunatic to spend a considerable portion of their life in his company. He is an evil, but interesting, person.

### The Ideal of Cloistered Seclusion.

For those who like a quiet and meditative life an appointment in an isolated asylum should approach the ideal of cloistered seclusion. The routine work can be got through, as a rule, in a comparatively short time and a large part of the day can, therefore, be given up to study. Some may prefer to put in an extra hour or two of sleep, or they may fall into vicious habits—there was one medical officer who read his morning paper through from the first page to the last, advertisements and all! Others, however, take to the arid study of philosophy and make themselves acquainted with everything from Aristotle to Bergson. As this does not in any way impinge upon their practice it does no harm and serves to keep the enthusiast from grumbling about diet and pay. Again, the history of insanity in regard to the views respecting it in former times and the treatment accorded to the insane; the influence of disordered mental states upon literature and history; or the relationship of criminality to mental defect and insanity; any one of these subjects, given the necessary intellectual interest, will tend to obviate boredom and may even lead to something of practical utility. Some new system of psychological investigation may even be excogitated; and another panacea for mental ills may be discovered. But, seriously, there are few kinds of medical work which allow such favourable opportunities for reading, either with some definite end in view or simply discursively and for amusement. And the literary output demonstrates this, as a study of the pages of the *Journal of Mental Science*—the quarterly publication of the Medico-Psychological Association—or of some of the other medical and scientific papers, will soon make clear.

### A Pleasant Backwater.

To sum up, the prospects financially, while not roseate, are steady and assured; the work is not exhausting, and it is interesting from many points of view, but there is little scope for initiative and little encouragement to individual effort. On the other hand, there is hope for an improvement in the conditions of asylum life generally. The trend is upwards, and possibly, in the not too distant future, the career of the asylum medical officer may be regarded as less of an "act in a backwater" than it is at the present time.

